# Long-term sugar consumption alters extracellular dopamine and acetylcholine levels in the nucleus accumbens shell

**DOI:** 10.3389/fnbeh.2026.1938066

**Published:** 2026-09-29

**Authors:** Gabriela Vera-Rivera, María José Olvera, María Isabel Miranda

**Affiliations:** Departamento de Neurobiología Conductual y Cognitiva, Instituto de Neurobiología, Universidad Nacional Autónoma de México, Juriquilla, Querétaro, Mexico

**Keywords:** appetitive learning, familiarity, rewarding, sensitization, taste

## Abstract

High dietary sugar consumption is associated with an increased risk of various diseases, making it imperative to identify the neural, nutritional, and behavioral mechanisms that underlie dietary habits to better understand and improve health. However, little is known about the neural mechanisms underlying prolonged consumption, making it difficult to determine the alterations caused by chronic sucrose intake in reinforcement and memory pathways, including the nucleus accumbens. Previous work in rodents has shown that long-term sugar consumption alters appetitive relearning, potentially leading to increased intake due to a disability in learning new negative consequences associated with the same taste, which possibly involves the mesolimbic dopaminergic system as well as cholinergic signaling. The objective of this study was to evaluate the extracellular levels of dopamine (DA) and acetylcholine (ACh) in the nucleus accumbens shell (NACsh) of adult male Wistar rats during novel (SN), familiar sugar consumption (2 days, SF), and after exposure *ad libitum* for 14 days to a 10% sugar solution (high-familiar groups, SH). Subsequently, on days 19–21, rats were habituated to the microdialysis chamber; on days 22 and 23, microdialysis was performed during consumption of novel, familiar, or highly familiar sugar. HPLC-EC microdialysis analysis revealed significant differences in the extracellular levels of DA and ACh in the NACsh between the highly familiarized and mildly familiarized groups (SF), and the group exposed to novel sugar. Specifically, baseline NACsh-ACh levels in the SH group were significantly higher and decreased during sugar consumption, whereas the novel sugar group showed a significant increase in ACh during and after sugar consumption; the SF group showed no change in ACh levels. In contrast, DA levels increased significantly during familiar sugar consumption (SF), but there were no changes after long-term consumption (SH) or during novel sugar (SN) consumption. The results demonstrate changes in cholinergic and dopaminergic signaling in the NACsh that negatively correlate with increased familiarity of a highly reinforcing stimulus, such as sugar. Specifically, the decrease in ACh and DA release following high-sugar familiarization suggests neuroadaptation in the NACsh, which could correlate with the inability to form new aversive associations with sweet flavors.

## Introduction

High dietary sugar consumption is associated with an increased risk of various diseases, making it imperative to identify the neural, nutritional, and behavioral mechanisms that underlie dietary habits to better understand and improve health. However, little is known about the neural mechanisms underlying prolonged consumption, making it difficult to determine the alterations caused by chronic sucrose intake in hedonic, homeostatic, or reinforcement memory pathways (Johnson et al., 2009; Malik et al., 2010; Imamura et al., 2015; Rippe and Angelopoulos, 2016). In this regard, various studies implicate the mesolimbic dopaminergic system in facilitating the rewarding and motivational aspects of consumption. Dopaminergic signaling in the nucleus accumbens (NAC) is fundamental for the associative learning of the reward value of highly palatable foods, such as sugar (Ågmo et al., 1995; Norgren et al., 2006; Meffre et al., 2019; Bobadilla et al., 2020; Gutierrez et al., 2020; Morales and Berridge, 2020; Salaya-Velazquez et al., 2020; de Sa Nogueira et al., 2021; Gutierrez and Simon, 2021; Hamelin et al., 2022). For example, oral sucrose stimulates dopamine (DA) release in the NAC, and pharmacologic or chemogenetic inhibition of NAC DA receptors reduces *libitum* sucrose intake in rats (Hajnal and Norgren, 2001). In general, the NAC has been shown to facilitate optimal reward prediction during motivational behavior; however, the precise role of its primary subregions, core (NACc) and shell (NACsh), in the recognition memory of familiar hedonic stimuli may differ, since these subregions diverge in their afferent and efferent connections (Heimer et al., 1991; Zahm and Brog, 1992; Jongen-Relo et al., 1993; Zahm and Heimer, 1993; Corbit et al., 2001; Hajnal and Norgren, 2001; Ikemoto, 2007) and subserve different functional properties (Carelli, 2004; Kelley, 2004; Saddoris et al., 2013; Zorrilla and Koob, 2013; Castro et al., 2015; Saddoris et al., 2015a). Among these functions, the NACc and NACsh appear to encode different aspects of value-based decision-making. In support of this, studies have shown that rapid DA release in the NACc can bias encoding toward subjective preferences in tasks involving risk, effort, and temporality (Day et al., 2010; Sugam et al., 2012; Saddoris et al., 2015b). Conversely, DA activity in the NACsh appears to encode reward outcomes: signals predicting larger rewards, compared to smaller ones, cause greater activation of dopaminergic cells and greater DA release in the cerebral cortex (Tobler et al., 2005; Roesch et al., 2007; Beyene et al., 2010; Nasrallah et al., 2011; Stopper and Floresco, 2011). Particularly, experiments using intraoral infusion on rats demonstrated that DA activity in the NACsh is related to motivational valence and novelty of stimuli, and that more generic motivational value could be related to NACc and prefrontal cortex activity (Bassareo et al., 2002; Norgren et al., 2006; Minnaard et al., 2022). Furthermore, studies have shown that DA-D1 receptors in the NACsh and in the amygdala are crucial for emotional processing during memory formation (Borja et al., 2020). This evidence suggests that dopaminergic activity in the NACsh could modulate the encoding of hedonic flavors, such as sugar, differently depending on the degree of familiarity, as well as its function during the expression of appetitive/hedonic memory.

Remarkably, studies have shown that insulin excites striatal cholinergic interneurons and increases DA release at dopaminergic terminals. Specifically, insulin facilitates the release of DA and glutamate in the NAC, thereby regulating appetite motivation, the hedonic response to glucose, and flavor-nutrient learning (Stouffer et al., 2015; Woods et al., 2016; Fordahl and Jones, 2017; Fetterly et al., 2021; Carr and Weiner, 2022; Finnell and Ferrario, 2022). In this regard, manipulation of insulin impacts NAC-DA release, which affects the hedonic value of glucose but not saccharin, suggesting a selective impairment of cholinergic/insulin signaling in the NAC (Woods et al., 2016; Carr et al., 2023) since cholinergic interneurons in the NAC stimulate DA release via axonal nicotinic receptors (Cachope et al., 2012; Threlfell et al., 2012; Liu et al., 2022). Furthermore, this evidence indicates that after long-term high-calorie consumption, dopaminergic and cholinergic activity in NACsh is altered and may be related to flavor-appetitive learning, leading to a significant increase in preference for higher glucose concentrations. This could have translational implications, including exacerbating weight gain and metabolic morbidities.

In this regard, we previously found that sugar was similarly preferred after three acute presentations or after 14 days of continued sugar consumption, and that NAC-induced dopaminergic disruption (via D2 receptor antagonism) differentially affects aversive association after acute or long-term sugar consumption and indicates that NAC DA-receptor activity has a differential function depending on the degree of sugar familiarity.

Taken together, the evidence suggests that long-term consumption of appetitive foods, i.e., sugars, could trigger escalated consumption through alterations in new learning involving changes in cholinergic and dopaminergic NAC function, with core and shell nuclei potentially differentially involved. Although previous studies link the NACsh to specific aspects of decision-making after learning, and DA release in the NACsh plays a differential role during cue-signaled and tracking information related to reward value (Sackett et al., 2017), to our knowledge, no studies have directly compared DA activity in the NACsh subregion during appetitive memory of different degrees of familiarity. Furthermore, reports of changes in cholinergic activity in the NAC are very limited, and it is unknown whether ACh levels in NACsh change following sugar consumption and after long-term sugar familiarization.

Here, we used microdialysis in freely moving rats to examine extracellular DA and ACh levels in the NACsh during consumption of novel, familiar, and highly familiar sugar. Thus, rats were first exposed to a sugar solution or permanently exposed for 2 or 14 days. We evaluated the impact of sugar familiarity on appetitive learning and its correlation with NACsh, DA, or ACh extracellular levels during consumption.

## Materials and methods

### Animals

A total of 35 male Wistar rats weighing 280–300 g at the beginning of the experimental procedures from the breeding colony at the Institute of Neurobiology (INB), UNAM, were used and handled in accordance with Mexican laws on animal care (Norma Oficial Mexicana SAGARPA) and the relevant regulations set forth by the Mexican Ministry of Health. The experimental protocol was approved by the Bioethics Committee of the INB-UNAM and complied with the National Institutes of Health “Guidelines for the Care and Use of Laboratory Animals” (NIH Publications No. 80-23, revised 1996). Rats were housed individually and maintained at 23 °C under an inverted 12-h/12-h light–dark cycle (lights off from 7:00 a.m. to 7:00 p.m.). All behavioral protocols were implemented during the dark phase of the cycle. Food and water were available *ad libitum* until the behavioral procedures began.

### Behavioral procedures

At the start of the behavioral procedures, the rats were divided into groups based on their familiarity with sugar: novel (SN) *n* = 9, familiar (SF) *n* = 10, or highly familiar (SH) *n* = 10.

All groups were subjected to the following long-term liquid intake protocol: Rats were exposed to 21 days of consumption of (a) tap water (SN and SF group) or (b) 10% sugar water solution (commercial sugar cane (*Zulka*®), composed of sucrose 99.9%) (SH group) as a single liquid *ad libitum* (Figure 1A). During the first 14 days, body weight, food pellet weight, and liquid intake consumed per day were measured (data not shown). Preliminary results from our laboratory showed that rats significantly prefer sugar over water, with insignificant daily water intake (<5%) (Vera-Rivera et al., 2020; Miranda et al., 2021). Thus, liquid consumption was measured by placing one bottle (sugar solution or water) on the home cage lid for 14 days. The glass bottles (500 mL) were refilled every 2 days with liquid (total weight = 500 g of liquid and bottle). Every day, at the same time, the bottles were weighed, and the daily liquid consumption (“grams”) was registered. The same amount of food pellets, 200 g (Lab Diet® 5001 Rodent Diet), was provided daily, with pellets weighed every 24 h. Also, rats were handled daily during the body weight procedure, allowing them to become habituated to the experimenter.

**Figure 1:**
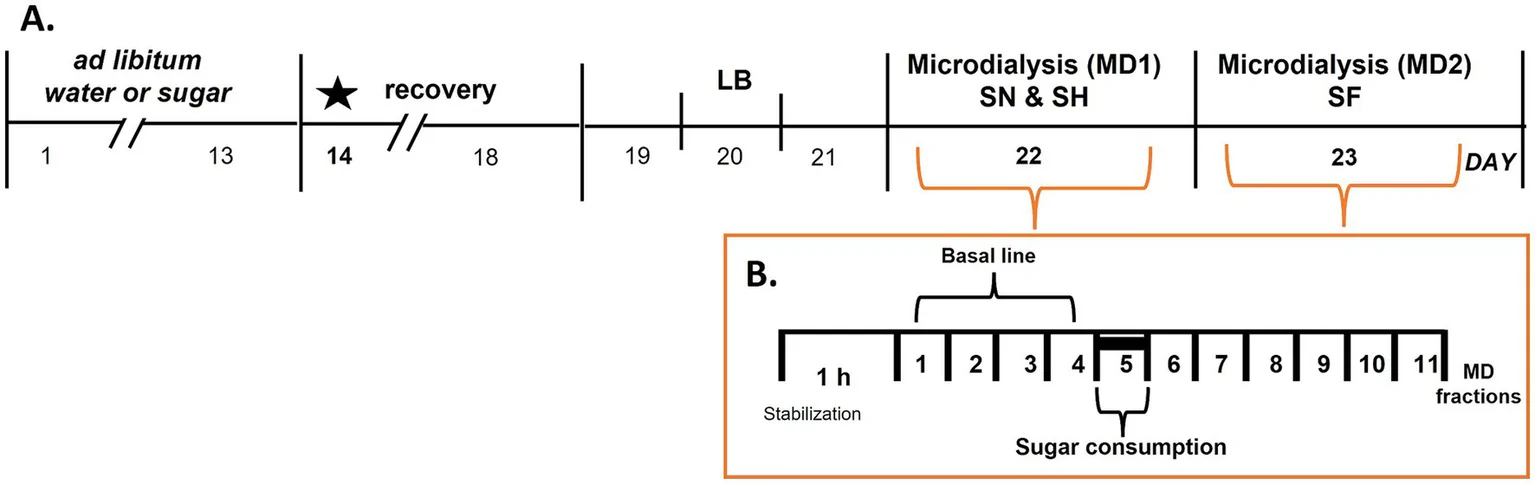
Experimental design for consumption **(A)** and microdialysis **(B)**. Exposure to sugar or water (days 1–13), surgery (star) and recovery of cannula implantation surgery (water or sugar ad libitum; days 14–18), liquid consumption in the microdialysis (MD) chamber (LB, days 19 and 21), microdialysis novel sugar (SN) and high familiar (SH) (days 22), and familiar (2 days, SF) group (day 23). A total of 11 sample fractions were collected on each MD sampling day after 1 h of stabilization, following probe insertion into the guide cannula. MD fractions 15 min, 2 µL/min.

### Surgery MD

On day 14, after the last record of fluid consumption, rats were anesthetized with an intraperitoneal (i.p.) injection of Zoletil 100/Xylazine 2% (1 mg/kg). They were placed in a stereotaxic frame, and a unilateral guide microdialysis cannula (Amuza-Eicom CXG-12, 0.40 mm ID/ 0.50 mm OD) was implanted in the NACsh (coordinates: AP + 1.8 mm, L + 0.9 mm, and V –6.2 mm from bregma) (Paxinos et al., 2014). The microdialysis guide cannula was fixed to the skull with two surgical screws and dental acrylic cement. The stylet was inserted into the guide cannula to prevent clogging. Tramadol (100 mg/1 mL) was administered orally. The general health status of the rats was monitored daily from days 15 to 21 (Figure 1A). *Ad libitum* access to liquid and food was maintained, except that rats exposed to sugar were also provided with a water bottle.

### Microdialysis

After the 5-day recovery period following stereotactic surgery (days 14–18), the animals were deprived of fluid for 18 h. They were accustomed to drinking water or sugar in a graduated bottle for 15 min/d for 3 days (LB, days 19–21) in the microdialysis chamber. Two microdialysis trials were performed; the first one was the day after the last chamber habituation session on day 22. SN groups were exposed to a sugar solution for 15 min for the first time during the first microdialysis (MD1, first novel taste presentation). The SF groups were exposed to the sugar solution in the microdialysis chamber the following day (on day 23) /15 min after the first microdialysis (MD2, second taste presentation). The SH groups were exposed to the sugar solution in the microdialysis chamber on day 22 of permanent sugar consumption (Figure 1A). The microdialysis procedure was performed according to a previously described protocol (Miranda and Bermudez-Rattoni, 1998; Miranda and Bermúdez-Rattoni, 1999). Microdialysis was started by connecting the probe (Amuza-Eicom CXI-12-03, 0.40 mm ID/ 0.50 mm OD, membrane length 3 mm) inlet to a microinjection pump system (Marca Bioanalytical Systems Inc. model MD-1020) that circulated the dialysis probe continuously at a rate of 2 μL/min with Ringer's solution for the analysis of acetylcholine (118 mM NaCl, 4.7 mM KCl, 2.5 mM CaCl_2_, and 10 μM neostigmine to prevent ACh degradation) and modified Ringer's solution for DA (147 mM NaCl, 2.7 mM KCl, 1.2 mM CaCl_2_, 1.0 mM MgCl_2_ pH 5.1) (Kurling-Kailanto et al., 2010). Then the microdialysis probe was inserted into the unilateral NAC guide cannula, the perfusion exudate from the first 60 min was discarded, and then samples were collected every 15 min thereafter. A total of 11 samples were collected on each microdialysis sampling day. In the fifth fraction of microdialysis, access to sugar consumption was provided. An additional six samples were collected before the microdialysis procedure was concluded (Figure 1B). All microdialysis samples were immediately frozen at −30 °C and stored until analysis by high-performance liquid chromatography (HPLC).

### Acetylcholine quantification

Extracellular levels of ACh in microdialysis samples were quantified by HPLC (BASi PM-80, West Lafayette, Indiana, USA), with a BASi CC-SE injector (20 µL loop) and electrochemical detection (Epsilon), as described previously (Miranda and Bermúdez-Rattoni, 1999). Briefly, each dialysate sample was assayed using an ACh/choline chromatographic assay kit (BASi) consisting of an analytical polymeric reversed-phase column (BASi MF-6150) and an ACh/choline immobilized enzyme reactor (IMER, BASi MF-6151). The mobile phase (flow rate, 1 mL/min) consisted of a 50 mM sodium phosphate buffer, pH 8.5, supplemented with 0.05% Kathon reagent (BASi), a broad-spectrum antimicrobial suitable for enzyme preservation. ACh was separated in the analytical column and hydrolyzed into acetate and choline by acetylcholinesterase in the IMER. The choline product was then oxidized by choline oxidase into betaine and hydrogen peroxide. Hydrogen peroxide was detected electrochemically by a platinum working electrode at + 500 mV with an Ag/AgCl reference electrode. The limit of detection was ∼0.1 pmol, and the signal-to-noise ratio was >2. A linear regression curve was made using ACh standards, and the compound peak areas for the samples were compared with those of the standards. ACh levels were calculated as pmol/20 μL.

### Dopamine quantification

Extracellular levels of DA on the microdialysis samples were assayed also by HPLC (BASi PM-80, West Lafayette Indiana, USA), injector (BASi CC-SE, 20 µL loop) with a stationary phase or reversed-phase C18 column (Syncronis 250 x 4.6 mm, 5 µm particle size, Thermo Scientific) at 30 °C and an electrochemical detector (Epsilon) with a carbon electrode; the potential was set to + 680 mV versus a reference electrode (Ag/AgCl) at a sensitivity of 5 nA. The mobile phase consisted of 90 mM monobasic sodium phosphate (NaH2PO4), 50 mM citric acid (C6H8O7), 1.7 mM sodium octyl sulfate (C17H35NaO4S), and 0.5 mM EDTA, adjusted to pH 3 with 17% (v/v) methanol. The flow rate was maintained at 1 mL/min at a pressure of 1500 psi. A standard curve was prepared using known concentrations, and the peak area was recorded, analyzed, and integrated for each chromatogram with Chrom Graph Report v.2.3 software (Bioanalytical Systems, Inc.). DA levels were calculated as pmol/20 μL.

### Histological analysis

Upon completion of the microdialysis trials, rats were overdosed with sodium pentobarbital and transcardially perfused with 0.9% saline. The brains were removed and placed in a 10% formaldehyde solution for 4 days, after which they were immersed in a 30% sucrose solution. Coronal sections (40 μm thick) were cut and stained with cresyl violet. Sections were observed under the microscope to determine correct cannula placement (Figure 2). Six rats with evidence of lesion at the site of interest (NACsh) or with a misplaced guide cannula were excluded from further analysis.

**Figure 2:**
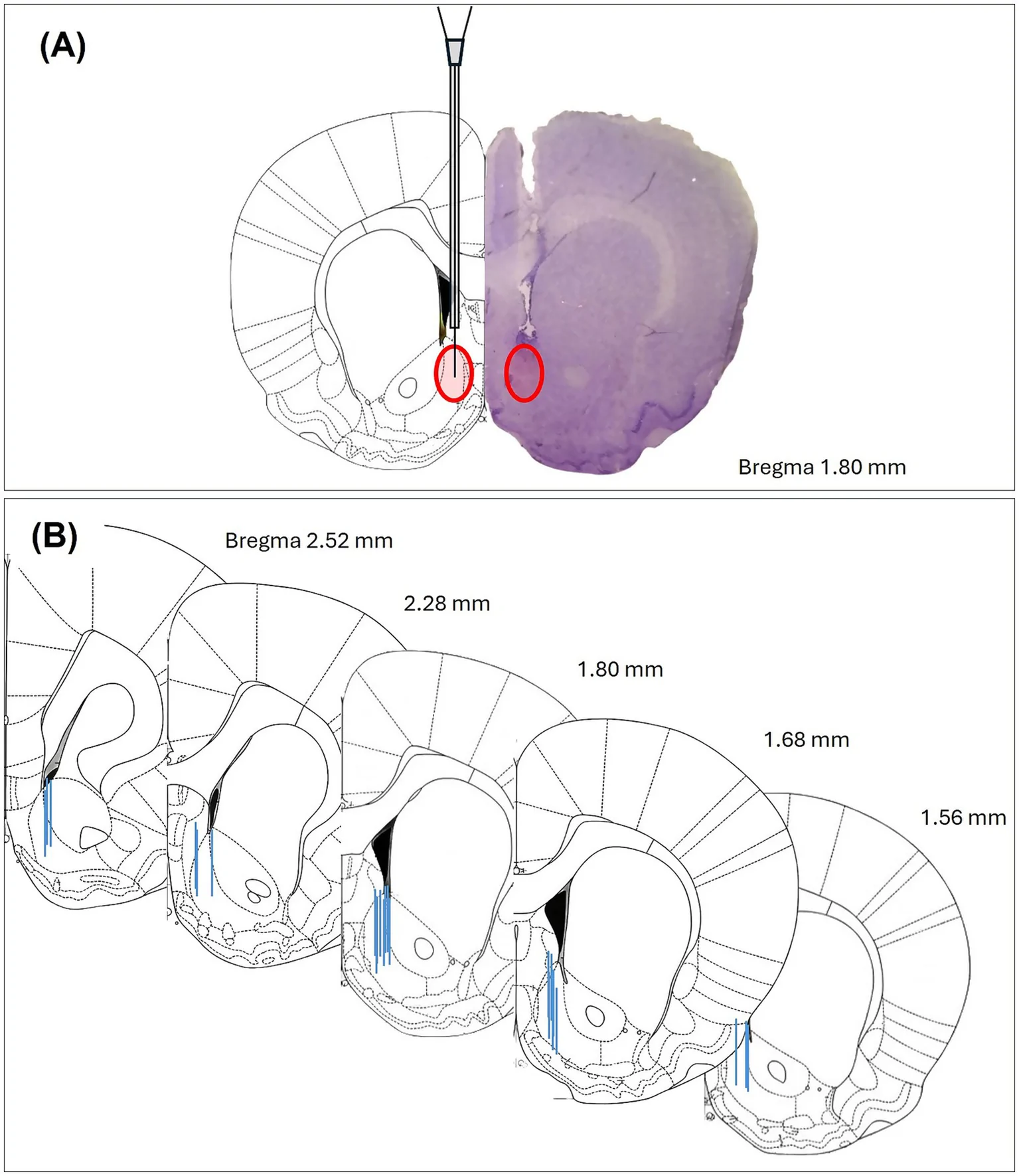
**(A)** Coronal section diagram and representative photomicrograph of cannula and injector (cresyl violet), and schematic drawing depicting the approximate unilateral probe site in the right nucleus accumbens shell (NACsh) (oval) (modified from Paxinos et al., 2014), **(B)** coronal section diagrams indicating, with lines, the locations of probe sites.

### Statistical analysis

Figures and statistical analysis were performed using GraphPad Prism 9 software (Systat Software, San Jose, CA). Data for each variable were presented as mean ± standard error of the mean (SEM). Statistical differences were evaluated using repeated-measures analysis of variance (rmANOVA) to compare all microdialysis fractions. These were followed by pairwise multiple comparison procedures (Fisher's post-hoc test). Finally, a paired *t*-test was performed to compare the differences within each group over the course of the microdialysis. To compare sugar intake volumes, a one-way ANOVA was performed. *p* values < 0.05 were considered significant.

## Results

### Prolonged sugar consumption does not lead to increased body weight

The rats' body weight in the SH groups showed a similar gain to that of control animals over the following 14 days. The similar body weight values obtained replicate previous experiments of our laboratory (Vera-Rivera et al., 2020), showing that prolonged exposure to sugar reduces pellet consumption by increasing sugar water intake, suggesting a compensatory mechanism to maintain stable calorie intake, which is consistent with previous research (Caynas-Rojas et al., 2019; Vera-Rivera et al., 2020).

### Cannula and probe placement verification

Only rats confirmed to have their guide cannulae and/or microdialysis probe in the NACsh were included in the data analyses (Figure 2). Two (6.8%) cannulated rats were excluded due to misplacement of cannulae/injectors or the probe.

### Sugar consumption during microdialysis

Sugar consumption volume was measured during the 5th microdialysis fraction. To compare intake groups, a simple ANOVA was performed on total volume (mL). As shown in Figure 3 (inserts), no significant differences among ACh analysis groups were found in sugar intake, as revealed by ANOVA (F2,9 = 2.262; *p* = 0.16), despite a tendency toward increased consumption in the high familiar group (SH). Similarly, no differences in consumption were found in the groups evaluated for DA (F2,14 = 1.647; *p* = 0.22).

**Figure 3:**
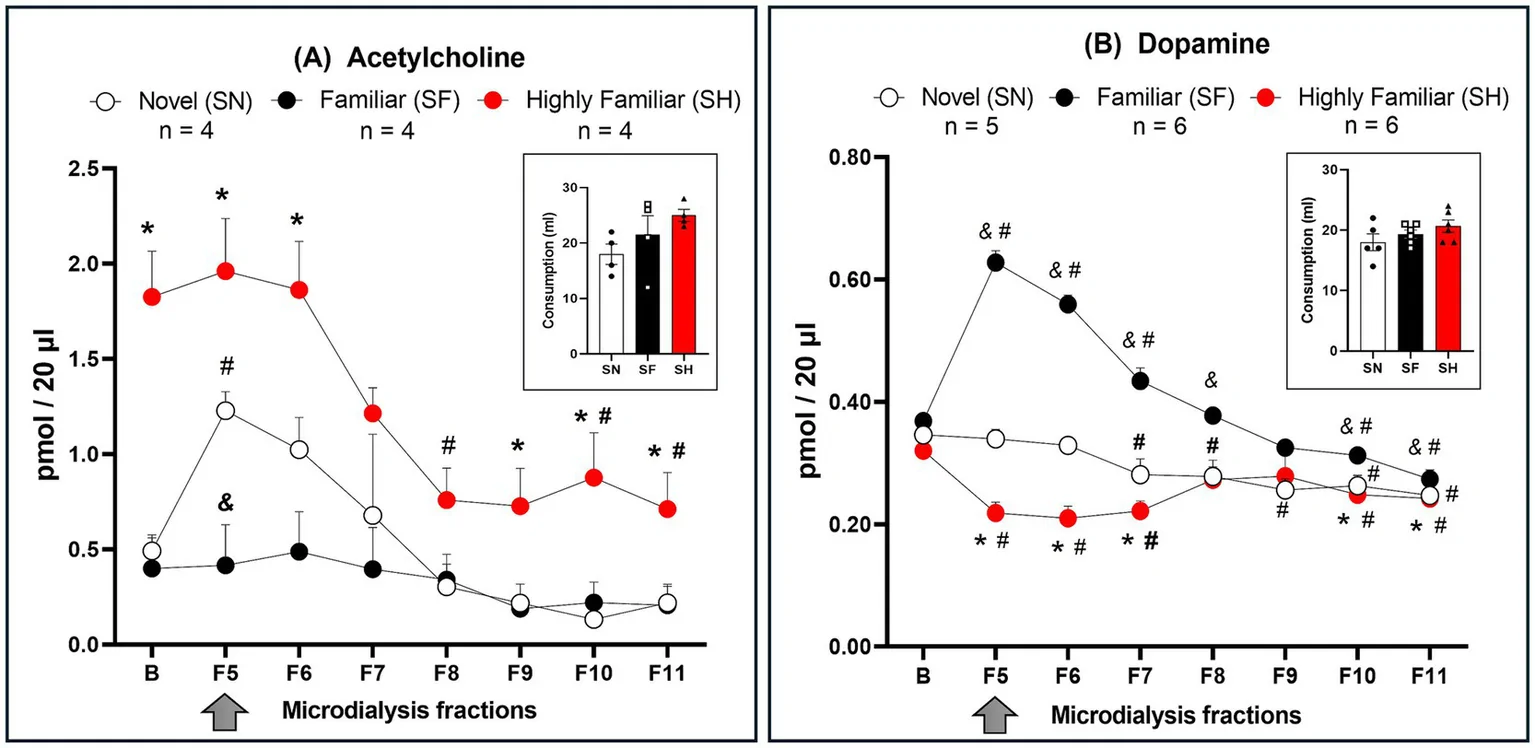
Extracellular levels of acetylcholine **(A)** and dopamine **(B)** in the NACsh. Baseline “B” levels are the average of the first four fractions of microdialysis after stabilization. *p < 0.05 SN vs. SH; and < 0.05 SN vs. SF; # *p* < 0.05 B vs. same group fractions. Inserts show the volume consumed (mL) during fraction 5 (arrow). Data are presented as mean values ± SEM.

### Acetylcholine levels are increased significantly after high familiarity with sugar, but decrease during consumption

Figure 3A shows the ACh levels in the NACsh throughout the baseline (average of the first 4 fractions, B) and the posterior 7 microdialysis fractions. Repeated measures ANOVA of ACh levels in NACsh (Figure 3A) showed significant differences between groups (F2,8 = 16.549; *p* = 0.0014) between fractions (F7,14 = 13.468; *p* < 0.001); and significant interaction (F14,56 = 2.827; *p* = 0.003). Post hoc Fisher’s analysis showed significant differences between groups on basal fractions as well as fractions 5–7, 9–11 (*p* < 0.5). ACh basal levels were significantly higher in the SH group than in the SN and SF groups (*p*, with a significant decrease in extracellular ACh from F7 to F11 (paired *t*-test, *p* < 0.05). After consumption, SH maintained higher ACh levels than SN and SF, despite their ow < 0.001); during sugar consumption (F5), the SH group also differed significantly from the other groups (*p* < 0.05). Furthermore, the SN group also differed significantly from the SF group (*p* < 0.05). Only the SN group showed a significant increase in ACh during sugar consumption (F5), recovering to baseline ACh levels from fraction 6 onward (paired *t*-test, *p* < 0.05). This elevation in baseline ACh levels was not observed in the familiar sugar group, which showed no change from baseline in any subsequent fractions (F5–F11) (paired *t*-test, *p* > 0.05). Conversely, the high-familiar sugar (SH) group showed significantly higher extracellular levels relative to the baseline fraction, which did not change until 30 min after sugar consumption decreases, being significantly higher than the SF group throughout fractions (F6–F11) (*p* < 0.05), as well as during the last two fractions in comparison to the SN group (F10–F11) (*p* < 0.05). These results demonstrate a differential basal cholinergic content in the NACsh depending on the magnitude of preexposure to sugar, as well as significant and opposite changes in extracellular ACh levels in the NACsh during and after sugar consumption, with respect to the degree of familiarity. Furthermore, the differential changes in baseline, during, and after sugar consumption observed across the three groups indicate the impact of different experiences prior to sugar consumption, where long-term previous sugar exposure significantly and differentially altered extracellular basal ACh levels in the NACsh.

### Dopamine levels increase significantly only during mild familiar sugar memory retrieval

Figure 3B shows the DA levels in the NACsh throughout baseline (average of the first four fractions, B) and the posterior 7 microdialysis fractions. Repeated measures ANOVA of the DA levels showed significant differences between groups (F2,14 = 25.747; *p* < 0.001), between Fractions (F7,14 = 28.468; *p* < 0.001), and a significant interaction (F14,98 = 25.426; *p* < 0.001). Post hoc Fisher’s analysis showed significant differences between groups for fractions 5–7 (*p* < 0.05), but no differences in basal DA levels and the last 4 fractions (F9–F11).

Basal DA levels were similar across all groups; however, during sugar consumption (F5), the SF group differed significantly from the other 2 groups (*p* < 0.001). Furthermore, the SN group also showed a significant difference with the SH group (*p* <.001). Still, only the SF group showed a significant increase in DA during sugar consumption (F5), which was maintained for the next 45 min (F6–F8) (paired *t*-test, *p* < 0.05), with DA returning to baseline from fraction 9 onward. This elevation in baseline DA levels was not observed in the SN group, which showed a slight decrease from baseline in the subsequent fractions (F7–F11) (paired *t*-test, *p* < 0.05). Similarly, the SH group showed a significant decrease in extracellular levels relative to the baseline fraction during sugar consumption and until 30 min after (F6–F7) (paired *t*-test, *p* < 0.05). After consumption, SH maintained lower DA levels than SN and SF, being significantly lower than the SF group throughout fractions (F6–F10) (*p* <.05), as well as during F6-F7 fractions in comparison to the SN group (*p* <.05). These results demonstrate significant and opposite changes in DA extracellular levels in the NACsh during and after sugar consumption, depending on the magnitude of preexposure to sugar.

## Discussion

The present results demonstrate differential changes in cholinergic and dopaminergic signaling (e.g., extracellular neurotransmitter levels) in the NACsh that negatively correlate with higher sugar familiarity. Measures of ACh in free-moving rats during sugar consumption demonstrate that after long-term consumption (21 days) of sugar as the only liquid available, the ACh basal level in the NACsh is significantly elevated in comparison to the levels observed in rats that were never exposed or had only one experience with sugar. However, these elevated ACh levels decrease significantly 30 min after sugar consumption and remain low for the next 2 h. This extracellular ACh change is not observed when sugar is “slightly” familiar (rats' second-time consumption). In this regard, sugar intake volumes did not differ significantly from those of rats that were familiarized (SF) or that consumed sugar for the first time (SN); however, intake increased significantly in highly familiarized rats (SH). In fact, acute consumption of familiar sugar (second time, 15 min/24 h) does not alter baseline extracellular ACh levels, which remain stable across all measurements. This stability in ACh levels contrasts with the significant elevation in ACh during the first (novel) sugar consumption. Groups acutely consuming sugar for the first time (SN) (15 min) exhibit a significant increase in ACh during consumption that persists for 30 min, with baseline levels recovering 1 h later.

ACh is released by local NAC interneurons (Meredith et al., 1989) and appears to have a modulatory effect on feeding behavior, since during free-feeding of pellets and water consumption, ACh increased at the end of a meal (Mark et al., 1992). Also, it has been shown that daily intermittent sucrose increases ACh levels in the NACsh, which occurred during the first sample after the sucrose meal ended (Rada et al., 2005). These data partially agree with our result but yield a different interpretation when variables such as deprivation and changes in eating patterns over 21 days are included in models that attempt to replicate binge-eating behavior, suggesting that increasing the activity of the cholinergic interneuron in the NAC reduces palatable food consumption, lending support to the hypothesis that ACh in the NAC influences motivation related to satiety signaling (Aitta-Aho et al., 2017). Furthermore, previous reports indicate that ACh nicotine receptors activate the mesolimbic DA system (Pontieri et al., 1996; Pich et al., 1997) and have been related to the enhancement or suppression of sucrose preference and intake following sucrose withdrawal (Kawamura et al., 2022), suggesting that nicotinic receptors are involved in decreased motivation for palatable foods (Donny et al., 2011).

Our results further support the idea that ACh in the NACsh plays an important role in flavor appetitive memory, modulating neurotransmission according to the degree of familiarity, which may signal changes in reinforcing and hedonic properties after long-term sugar consumption, since previous evidence indicates that these properties evolve with exposure time and context (Kawamura et al., 2022; Donny et al., 2011).

Specifically, the decrease in ACh and DA extracellular levels following high-sugar familiarization suggests neuroadaptation in the NACsh, which could be related to the inability to form new aversive associations with sweet flavors demonstrated in previous evidence (Vera-Rivera et al., 2020). Accordingly, after long-term high-calorie consumption, i.e., sugar, dopaminergic activity in NACsh is altered and may be related to flavor-appetitive learning, leading to a significant increase in preference for higher glucose concentrations. This could have translational implications, including exacerbating weight gain and metabolic morbidities. In this regard, it is important to mention the evidence of the effect of insulin on DA release that is blocked either by maintaining rats for six weeks with continuous access to a high-sugar, high-fat diet or by NACsh insulin inactivation, with the consequence of decreased glucose lick microstructure parameter as an indication of hedonic impact (Woods et al., 2016; Carr et al., 2023). This evidence is consistent with studies showing that insulin excites striatal cholinergic interneurons and increases DA release at dopaminergic terminals (Stouffer et al., 2015; Woods et al., 2016; Fordahl and Jones, 2017; Fetterly et al., 2021; Carr and Weiner, 2022; Finnell and Ferrario, 2022) suggesting a selective impairment of cholinergic/insulin signaling in the NAC (Woods et al., 2016; Carr et al., 2023) since cholinergic interneurons in the NAC stimulate DA release via axonal nicotinic receptors (Cachope et al., 2012; Threlfell et al., 2012; Liu et al., 2022). Further research is needed to establish, in simple sugar-familiarization models such as those used here, the differential involvement of nicotinic receptors in the NACsh to determine changes or neuroadaptations related to the degree of sugar or reinforcing food exposure.

Although the exact contribution of NACsh DA to reward and hedonic processing remains unclear, most researchers agree that it is involved in feeding behavior (Wiss et al., 2018). DA neurons respond to exposure to a novel food paired with a cue and, in a subsequent exposure, will not induce neuronal firing. In contrast, the cue activates DA neurons but not food alone, suggesting that DA neurons are involved in conditioned learning (Schultz, 2016). Interestingly, increased DA during eating highly palatable food wanes with repeated exposure, but learning about the stimulus amplifies DA transmission, extending it to predictive stimuli (conditioning) (Bassareo and Di Chiara, 1997). Appetitive food stimuli (in specific stimulus containers) induce an increase in DA transmission in the NACc but not in the NACsh, where DA release was inhibited; in other words, DA transmission in the NACsh was not activated by conditional appetitive stimuli, suggesting that the NACc, but not the shell, is a critical subregion for the motor expression of motivated responding (Bassareo and Di Chiara, 1999).

Although these differences between NAC subregions suggest that DA transmission plays different roles in motivated behavior and associative processes, they do not rule out its involvement in taste memory, depending on the degree of familiarity. The present results suggest that dopaminergic transmission in the NACsh may be related to the expression of appetitive memory for reinforcing stimuli learned without apparent contextual or aversive associations, and that this transmission is significantly reduced after high familiarization with the reinforcing hedonic stimulus (e.g., sugar).

Recent evidence shows that NACsh dopaminergic receptor agonism reduced the intensity of the innate appetitive response to novel sugar. However, the DA receptor agonism does not disrupt memory recognition once sugar becomes familiar or highly familiar, indicating that the dopaminergic balance in NACsh changes as a highly hedonic stimulus is experienced (Miranda et al., 2026). However, evidence of pharmacological manipulation of the DA system has led to contradictory results. On the one hand, DA injected directly into the NAC can increase feeding and liquid intake (Swanson et al., 1997). However, others have not been able to modify feeding behavior with specific DA agonists or antagonists (Baldo et al., 2002).

Taken together, numerous lines of evidence point to the importance of the NACsh in eating behavior; however, the variety of behavioral models employed, which include changes in context, different types of learning and conditioning, as well as implicit variables present in experimental designs, such as hunger, thirst, intermittent consumption, age, and sex, among others, complicates interpretation. Furthermore, it is important to note the potential differential NACsh function across different types of solid food, sucrose concentrations, and their combinations, which complicates the interpretation of dopaminergic and cholinergic activity during flavor-novel recognition and familiar sugar memory.

Here, the results demonstrate an increase in extracellular ACh, but not in DA levels, in the NACsh during the first encounter with a caloric and hedonic natural reinforcer. Sugar consumption occurred under free-access conditions and without contextual variables, with fluid deprivation as the only variable that could be added to the motivational response. Furthermore, the data show that after appetitive (unconditioned) sugar learning, once the sugar becomes familiar, ACh levels remain unchanged. In contrast, DA levels increase significantly during sugar memory recall, e.g., during the second acute presentation. These results indicate an opposite shift in the ACh/DA balance in the NACsh due to the formation of long-term (24 h) flavor appetitive memory. However, when sugar becomes highly familiar through daily, continuous consumption (21 d), baseline extracellular ACh levels increase significantly but decline during sugar consumption (i.e., during recall of the highly familiar memory). Furthermore, while baseline extracellular DA levels do not change after long-term sugar consumption, they decrease during memory recall of highly familiar sugar, suggesting negative regulation of cholinergic and dopaminergic transmission in the NACsh. Further experiments are needed to distinguish the effects of sugar memory familiarization from the broader effects of repeated sugar experience on other aspects, such as metabolism, context, and reinforcement learning.

In conclusion, this research demonstrates changes in cholinergic and dopaminergic signaling in the NACsh that negatively correlate with increased familiarity with a highly reinforcing stimulus, such as sugar. Specifically, the decrease in ACh and DA extracellular levels following high-sugar familiarization suggests neuroadaptation in the NACsh, which could correlate with re-learning and new associations with sugar.

## Data Availability

The raw data supporting the conclusions of this article will be made available by the authors, without undue reservation.
